# Monitoring rural-urban transformation in the coastal region of Rabat-Sale-Kenitra, Morocco

**DOI:** 10.1371/journal.pone.0290829

**Published:** 2023-08-31

**Authors:** Safia Loulad, Thanh Thi Nguyen, Mohamed Rabii Simou, Hassan Rhinane, Andreas Buerkert

**Affiliations:** 1 Geosciences Laboratory, Department of Geology, Faculty of Sciences, University Hassan II, Casablanca, Morocco; 2 Organic Plant Production and Agroecosystems Research in the Tropics and Subtropics, University of Kassel, Witzenhausen, Germany; Van Lang University: Truong Dai hoc Van Lang, VIET NAM

## Abstract

Worldwide urbanization drives rural-urban transformation (RUT) which has major consequences in many countries of the Global South where there is an urgent need to better understand and manage the underlying processes and consequences for ecosystem services. To fill existing knowledge gaps on the extent and time course of RUT in Morocco, this study focused on (i) analyzing the spatial patterns of rural-urban transformation in the Rabat-Sale-Kenitra (RSK) region from 1972 to 2020, (ii) identifying key mechanisms of change, and (iii) defining the main driving forces behind the spatial transformation patterns. To this end, we processed data of the Landsat free archive, historical grayscale Corona images, and nighttime lights datasets on Google Earth Engine (GEE) using machine learning classifiers and LandTrendr spectral-temporal segmentation algorithms. With an overall accuracy (OA) ranging from 88–95%, the results revealed that during the study period the RSK region experienced a 473% growth of horizontal built-up reflected in an area increase from 63.4 km^2^ to 299.9 km^2^. The main changes occurred along the Kenitra-Rabat-Temara axis and in central cities connected to the main road network. The horizontal expansion of large and medium-sized cities led to the formation of a Rural-Urban Interface (RUI) on the outskirts. The urban sprawl of some cities has affected the surrounding rural lands within the RUI. Environmental, social, economic, and political forces have interacted in shaping the changes in rural-urban landscapes.

## Introduction

Since their initiation >8000 years ago, human settlements have caused rural-urban transformation [1]. According to recent UN reports, over half of the world’s population currently lives in urban areas [2], a proportion forecast to increase to two-thirds by 2050 [3]. As a consequence of ongoing population growth and substantial rural exodus, the largest increase will occur in countries of the Global South [4, 5], leading to major effects on ecosystem services [6].

Although urbanization can be associated with many social and economic benefits [7], it may lead to population destabilization [8], profound impact on land use dynamics [9], and massive and uni-lateral use of natural resources [10], causing significant societal, environmental, and economic issues distortions [11]. This alters the shape, size, and structure of cities, beyond their formal administrative boundaries, contributing to land quality degradation [12, 13], and the conversion of agricultural lands [14]. Furthermore, it has blurred the boundaries between rural and urban areas [15], leading to the formation of a transition zone often referred to as the “rural-urban fringe” or the “rural-urban interface” (RUI). This interface has unique characteristics and an identity that is neither rural nor urban [16, 17]. As a “rurban” entity with new opportunities of “being” and “becoming” [18], it encompasses many of the gradual changes that occur in (formerly) rural areas as a result of the expansion of the urban core.

Remote sensing and Geographic Information Systems (GIS) have emerged as powerful tools for studying the ongoing evolution of urbanization and its spatial dimensions. These technologies offer a consistent and objective means of measuring landscape transformations over time [19–21]. However, the management of big data obtained from Earth Observation (EO) and the analysis of large-scale cases have presented challenges in previous studies due to certain soft- and hardware limitations [22].

In the last decade, the development of the Google Earth Engine (GEE) cloud computing platform has facilitated spatio-temporal analysis of landscape patterns by overcoming many of the existing limitations [23, 24]. The platform allows for faster analysis of geospatial big data of different formats and resolution allowing for time series analysis since 1975 [25–27]. All datasets, interfaces, and algorithms are easily accessible on the cloud allowing to perform multiple analyses across regional and global scales [22, 28].

Since 2018 many studies used have employed free GEE products in conjunction with classified satellite images for comprehensive LULC analysis on regional, continental, and global scales and over extended periods using advanced processing models and customized algorithms specifically designed for land cover classification and change detection, enabling the detection of fine-scale changes [22, 24, 29–32]. For instance, a recent study applied this approach to quantitatively assess global urban growth and green recovery patterns observed over the past three decades [33]. The authors utilized the Random Forest (RF) classifier, the LandTrendr temporal segmentation algorithm, and a fusion of Landsat series images from 1985 with open global urban-extent maps. The study yielded an overall accuracy ranging from 76% to 90% and provided valuable insights into global urbanization dynamics and ecological rehabilitation.

However, for Morocco most of such studies have focused on small-scale urbanization processes, with a particular emphasis on major metropolitan centers like Casablanca and Fez [34–38]. These studies have predominantly relied on classical processing methods and mainly examined land use and land cover (LULC) changes since 1984, when free Landsat images of 60m became accessible in Morocco. Little attention has been paid to the transformations that urbanization has triggered on the rural side and sustainability issues raised by the LULC changes detected [39]. Furthermore, the Rabat-Sale-Kenitra (RSK) region has not been specifically investigated within the scope of existing studies.

In our study, we monitor the spatial rural-urban transformation in the region of Rabat from 1972 to 2020, using a combination of high resolution black/white CORONA images from the 1970s, a series of Landsat images from 1985 to 2020, and recent Geospatial dataset accessible on GEE. We hereby aimed at (i) analyzing the spatial patterns of rural-urban transformation in the Rabat-Sale-Kenitra (RSK) region from 1972 to 2020, (ii) identifying key mechanisms of change, and (iii) defining the main driving forces behind the spatial transformations.

## Study area

**Fig 1:**
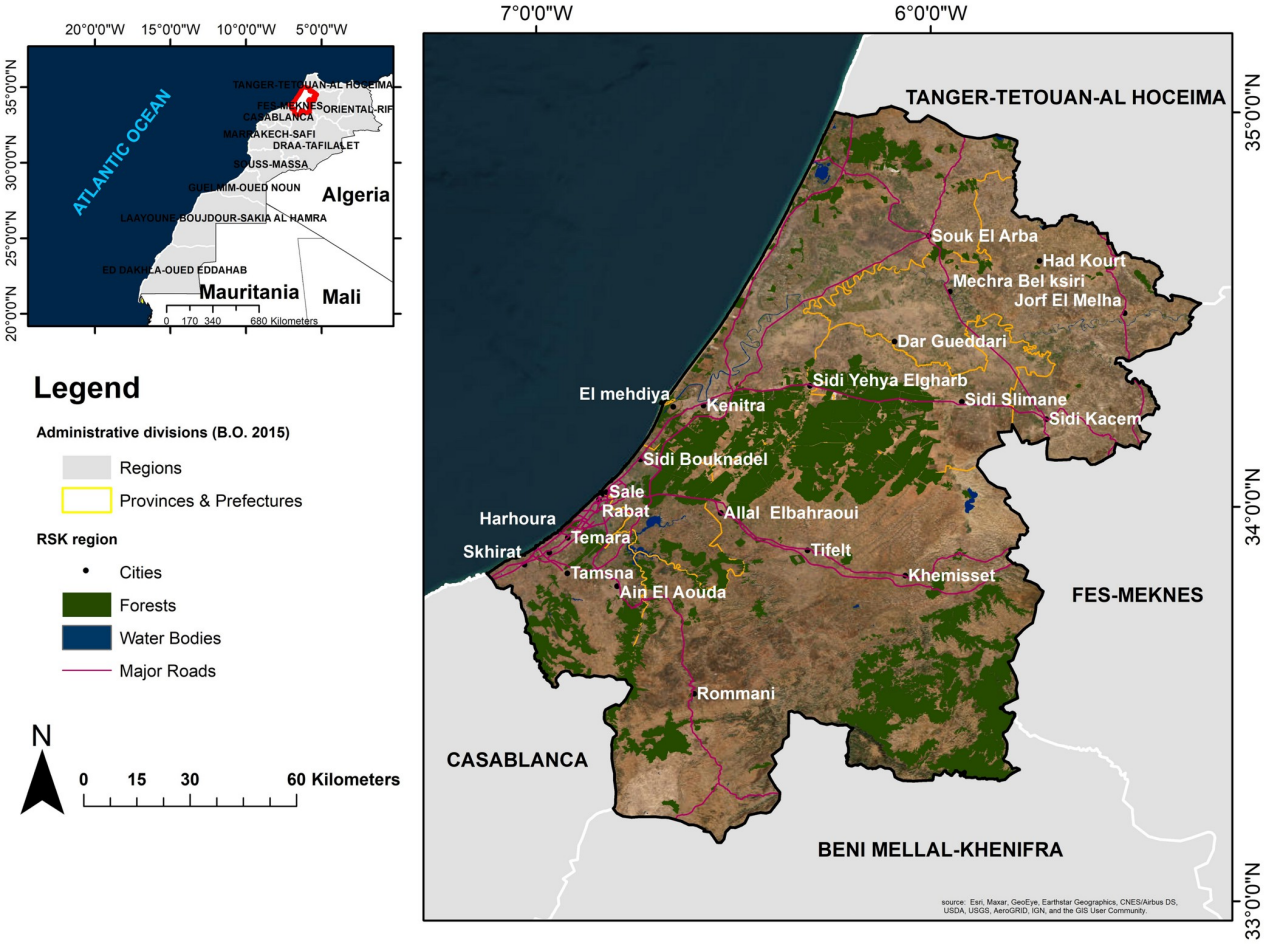
The geographic location and administrative boundaries of the study area. Map was created using ArcGIS (version 10.6) from Esri (http://www.arcgis.com). Basemap satellite images accessed from World Imagery Esri Tile Layer. Credits: Esri, Maxar, GeoEye, Earthstar Geographics, CNES/Airbus DS, USDA, USGS, AeroGRID, IGN, and the GIS User Community.

The RSK region, formed by the merger of the former Rabat-Salé-Zemmour-Zar and Gharb-Cherarda-Béni Hssen regions, is after the Casablanca-Settat region Morocco’s second most rapidly urbanizing area. It is the center of the country’s demographic, economic, and administrative activities, as it is home to the country’s administrative capital city [40]. RSK comprises the three prefectures Rabat, Salé, and Skhirate-Témara and four provinces (Kenitra, Khémisset, Sidi Kacem, and Sidi Slimane) (Fig 1). It encompasses 23 urban communes and 91 rural communes [41], with a total area of 18,194 km^2^ (2.6% of the national territory) and a population of 4,581,000 inhabitants [42] leading to a population density of 252 people per km^2^. It holds 12% of the country’s usable agricultural land (10,194 km^2^) and 4% of its total forest area (3513 km^2^) [40].

## Materials and methods

**Fig 2:**
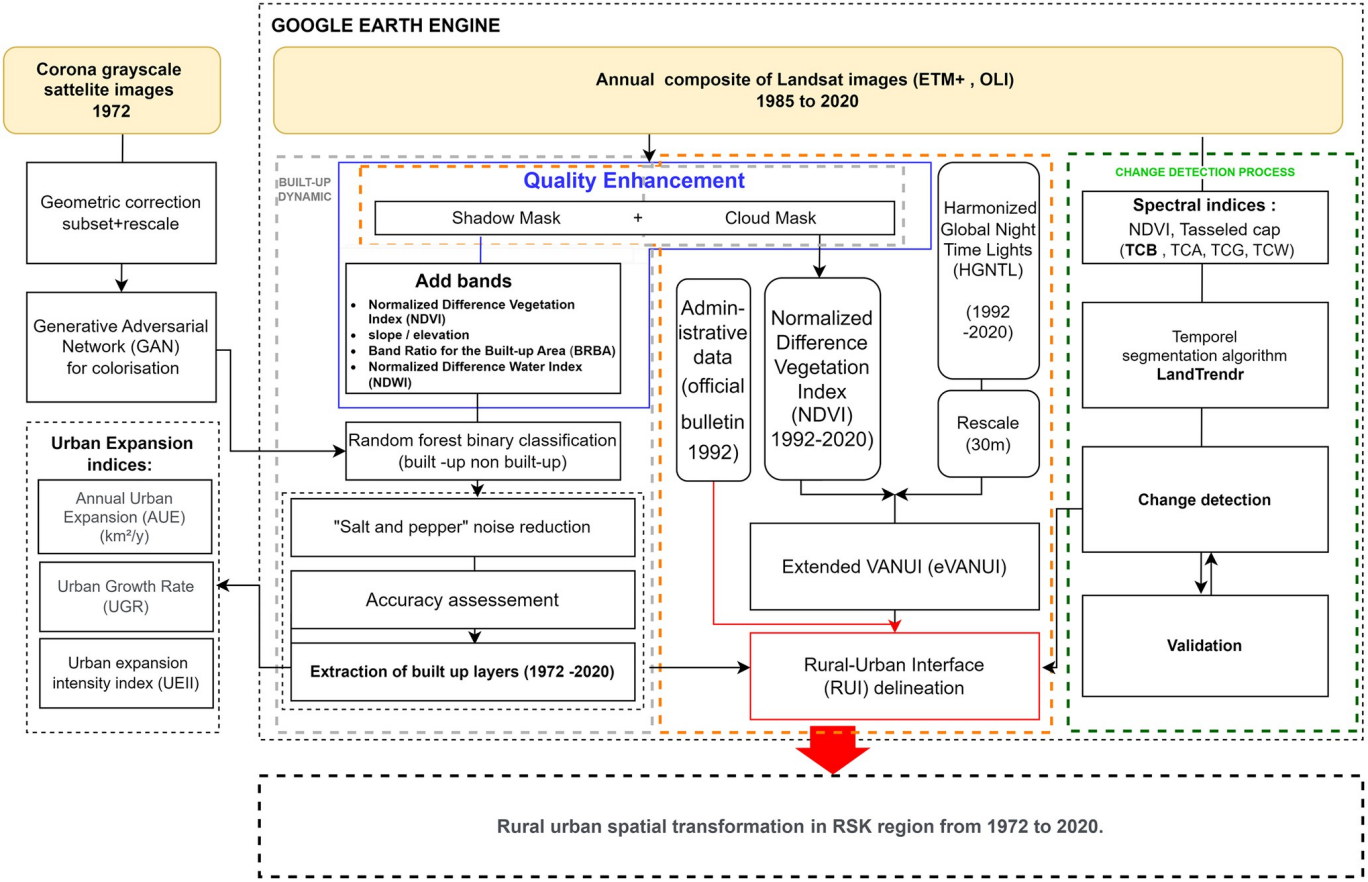
Flow chart of general framework analysis of rural-urban transformation.

To investigate the spatial transformation, we used data from Corona, Landsat 5, Landsat 8, and the Harmonized Global Night Time Light dataset (HGNTL) Table 1. Our study was carried out in three stages. First, we selected a black and white satellite images from the Corona KH-4B mission in 1972, taking into consideration its availability and the absence of cloud cover. Additionally, we chose Landsat free satellite images from 1985, which marked the availability of free Landsat 30m resolution data in Morocco, to study the urban fabric’s expansion over the 48 years from 1972–2020. Second, we combined the annual nighttime light product with the Normalized Difference Vegetation Index (NDVI) to delineate the limits of the RUI, which encompass the gradual changes that occurred in rural areas due to urban growth. Third, we employed a temporal segmentation approach to determine the spatial transformations in rural lands due to urban sprawl (Fig 2).

**Table 1:** Different datasets used in the study.

Acquisition year	Datasets	Mission (scenes)/sensor	Spatial resolution	Sources
**1972**	Corona	KH-4B-(DS1117-1011DF072—DS1117-1011DF086) 15 scenes	1.8m	U.S. Geological Survey. (USGS)–Declass 1
**1985,1995, 2005,2010, 1995,2000**	Landsat 5	Multi-Spectral Scanner (MSS)	30m	USGS
**2015, 2020**	Landsat 8	Operational Land Imager (OLI)	30m	
**1992–2020**	Harmoniz-ed Global Night Time Lights (HGNTL)	Defense Meteorological Program -Operational Line Scan System (DMSP/OLS) sensor / Visible Infrared Imaging Radiometer Suite (VIIRS)	~1km	National Oceanic and Atmospheric Administration (NOAA) / National Aeronautics and Space Administration (NASA). HGNTL Proposed by [43].

### Data pre-processing

#### CORONA images

**Fig 3:**
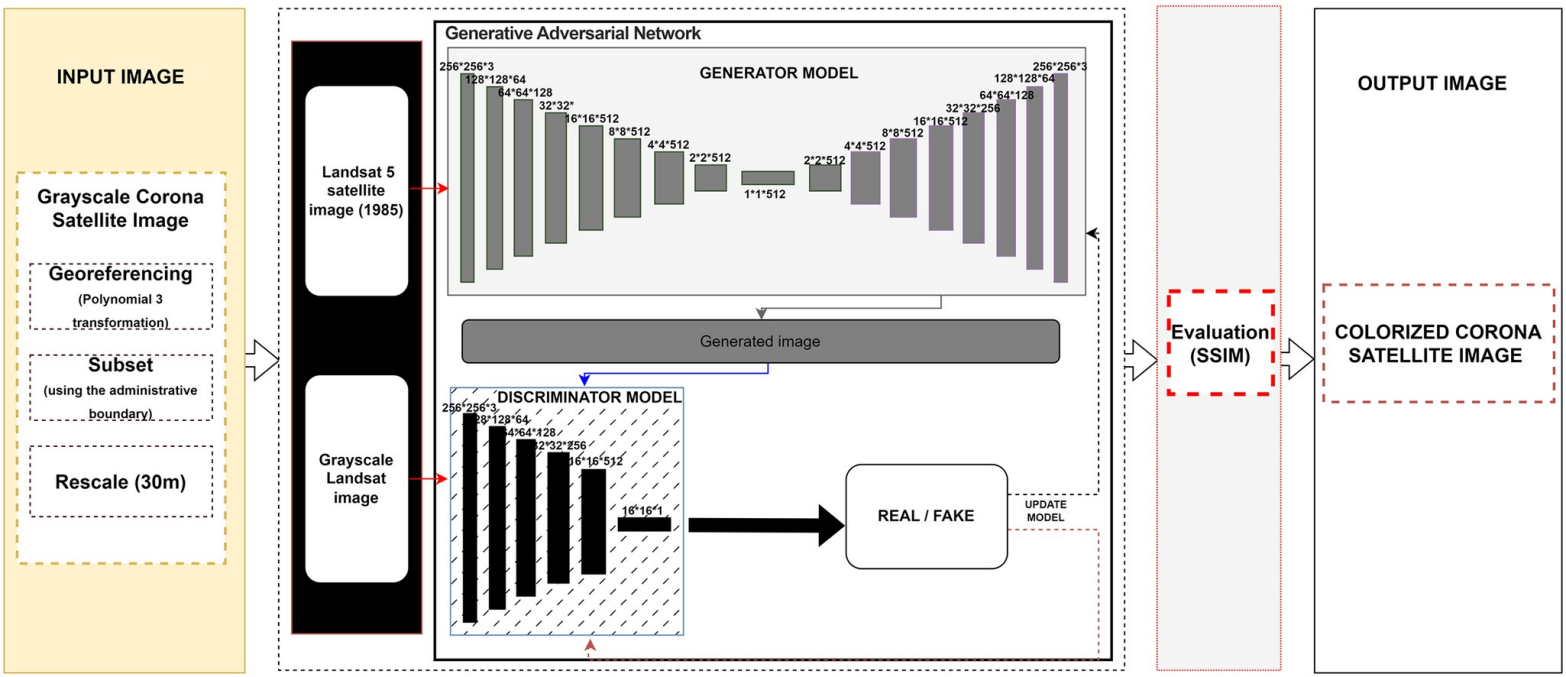
Architecture of the proposed GAN learning model used to colorize Corona grayscale images.

After georeferencing the Corona scenes using a third-order polynomial transformation, we mosaicked and rescaled the images to 30m to produce black-and-white images comparable with Landsat images S1a Fig. We then downloaded a 1985 Landsat 5 satellite scene from USGS, taken during the same season as the Corona image, and grayscaled it for colorization training using the Generative Adversarial Networks (GAN) deep learning approach. GANs consist of two main neural networks: a generative neural network (G) and a discriminative neural network (D; [44]). The generator (G) used the grayscale Landsat image to generate the true color information for each pixel, while the discriminator (D) compared the training results with the real data (the real RGB image). The training process continued until the GAN (D) was unable to distinguish between the real RGB image and the artificially colored Landsat image [45]; (Fig 3).

The results were evaluated using the structural similarity index (SSIM) Eq (1), which compares the pixel values of two images to determine the proportion of identical pixels and quantifies their similarity [44–46]. Upon achieving a SSIM >0.9, indicating a high level of similarity between the compared images, we applied the GAN training algorithm on the rescaled Corona image to produce the final colorized version S1b Fig.

$$SSIM(r,s)=\frac{\left(2\mu_{x}\mu_{y}+C_{1})(2\sigma_{xy}+C_{2}\right)}{\left(\mu^{2}{}_{x}+\mu^{2}{}_{y}+C_{1})(\sigma^{2}{}_{x}+\sigma^{2}{}_{y}+C_{2}\right)},$$ (1)

Where x represents the generated image, y is the RGB reference image, μ_x_ and μ_y_ are the averages of x and y, respectively. σ_x_^2^ and σ_y_^2^ represent the variance of x and y, respectively, and σ_xy_ defines the covariance between both generated and reference images. C_1_ and C_2_ are defined as (K_1_L)^2^ and (K_2_L)^2^, where K1 and K2 are fixed at 0.01 and 0.03 by default, respectively, and L is the dynamic range of the pixel values.

#### LANDSAT images

We created annual composites of the cloud/shadow-free Landsat images from 1985 by combining multiple reducer functions [47]. To improve the accuracy of land cover classification, we added several quality bands to the multispectral composite, including NDVI, the built-up area band ratio (BRBA), the normalized difference water index (NDWI) and the slope/elevation extracted from the ALOS DEM. All of these additional inputs were normalized to have the same scale as the satellite image (30m) Table 2.

**Table 2:** Quality bands added to the Landsat images.

Quality bands	Source	Equation	Resolution
**NDVI**	Landsat images	(Near infrared—Red) / (Near infrared + Red)	30m
**NDWI**		(Green—Near infrared) / (Green + Near infrared)	
**BRBA**		(Red / Shortwave infrared)	
**Slope/ Elevation**	ALOS DEM	-	

### Classification, smoothing, and validation

All images were processed in three stages. First, we employed the Random Forest (RF) meta-machine learning algorithm, provided by the Google Earth Engine (GEE) platform [48], to classify the pre-processed satellite images. The classification process involved the utilization of 2000 sampling datasets randomly distributed across the research area. These datasets were split into two random fractions, with 70% allocated for training and 30% for validation. The RF model was trained on 60 decision trees, enabling it to learn patterns and features within the images. The class chosen by the majority of trees becomes the model’s prediction [49]. According to [50], the RF classifier is one of the most accurate supervised classifiers because it uses multiple self-learning decision trees.

Second, we used a majority filter-based post-classification approach to improve the homogeneity and accuracy of the RF classification and reduce salt-and-pepper noise [51]. This technique replaces isolated pixels with the surrounding value and only displays the dominant classified pixels within a 3 x 3 pixel radius [52]. Third, we evaluated the classification performance using the remaining 30% sampling datasets (600; [53, 54]). This process involves comparing the classified values of the RF training model to those of the validation fraction to build an error confusion matrix, which is used to determine the overall accuracy (OA) [55]. According to [56], classification is considered accurate and useful for LULC studies when the OA is >85%.

### Urban expansion indices

We employed three main complementary growth indices to measure the rate, intensity and direction of urban expansion across the RSK region during the study period: the Annual Urban Expansion (AUE), the Urban Expansion Rate (UGR) and the Urban Expansion Intensity Index (UEII). These indices allowed us to determine the pace of the urban area evolution and identify the preference for urban growth as well as compare the local urban evolution pace with different countries.

First, we calculated the AUE (Eq (2); [57]) and the UGR (Eq (3); [58]) using the built-up area at two temporal periods (T1, T2).

$$\begin{array}{l} AUE=\frac{UA(T2)-UA(T1)}{T}, \end{array}$$ (2)

$$\begin{array}{l} UGR=\left(\frac{UA(T2)-UA(T1)}{UA(T1)}*\frac{1}{T}\right)*100, \end{array}$$ (3)

Where AUE is measured in square kilometers per year (km^2^/year), UGR is expressed as a percentage (%), UA(T1) and UA(T2) represent the built-up areas in the studied region at two temporal periods (T1) and (T2), and T represents the time interval between T1 and T2.

Secondly, we computed the UEII in the eight different geographical directions (i-th) out from the city center at two temporal periods T1 and T2 (Eq (4); [59].

$$\begin{array}{l} UEII_{i}=\left(\frac{UA_{i}(T2)-UA_{i}(T1)}{T*TLA_{i}}\right)*100, \end{array}$$ (4)

Where UEII_i_ represents the annual average urban expansion intensity index of the i-th spatial unit in a period (T), UA_i_(T1) and UA_i_(T2) denote the urban areas of the i-th spatial unit at time T1 (1972) and T2 (2020), respectively, and T represents the study period (48 years), TLA_i_ is the total area of the spatial unit i.

The UEII values are split as follows: 0 to 0.28 represents a slow expansion; 0.28 to 0.59 refers to low-speed expansion; 0.59–1.05 denotes medium-speed expansion; 1.05–1.92 means high-speed expansion; and >1.92 stands for very high-speed expansion [60].

### Change detection analysis

#### RUI delineation

To define the limits of the RUI, we employed a two-step process. Firstly, we merged two spatial products: the Harmonized Global Night Time Lights (HGNTL) collection (1992–2020) and the NDVI collection (1992–2020) to produce the eVANUI spectral index. This index maps the geographical boundaries between the functional area; including urban areas and suburbs [61], characterized by high socioeconomic activity and low vegetation cover indicative of ongoing urbanization, and the non-functional area, typically rural regions with lower socio-economic activity and higher vegetation cover.

Secondly, we used administrative data on the distribution of urban and rural communes in 1992 to exclude the RUI from the eVANUI-defined areas. This step aimed to accurately delineate the transitional zone between urban and rural domains without overlapping with purely urban regions as defined by administrative divisions.

The Vegetation Adjusted Nighttime Light Urban Index (VANUI) was proposed first by [62] as a way to reduce saturation in Nighttime Light (NTL) data from 1992 to 2013. Then it has been further developed and utilized in numerous studies, by incorporating additional data sources to address various research objectives. For instance, VANUI has been employed in studying carbon emission issue [63], and has been used also to reduce blooming effects and enhance the visibility of urban features [64].

In our study, we enhanced the VANUI index by integrating a harmonized NTL open product from 1992 to 2020 [43], which combines DMSP and VIIRS NTL datasets, along with NDVI collection from 1992 to 2020. Our primary goal was to leverage the inverse correlation between vegetation and night light intensity to accurately delineate the functional and non-functional areas.

To calculate the eVANUI index, we extracted the mean of the HGNTL collection and the median of the NDVI collection from 1992 to 2020. The mean of the HGNTL collection was employed to adjust for time-varying luminosity, while the median of the NDVI collection was used to reduce seasonal variability across vegetated land. Afterward, we rescaled the HGNTL to 30m. Finally, we reduced the value ranges of both products to (0,1) by removing all negative values for snow, water, and clouds from the NDVI S2a Fig, and by dividing all pixel values out of 63 on the HGNTL S2b Fig.

The eVANUI formula Eq (5) assigned low values to pixels with high vegetation cover and minimal luminosity (non-functional areas), and higher values to areas with high to medium luminosity and low to rare vegetation cover (functional areas). Then, the final delineation of both areas boundaries was achieved using a threshold-based method S3 Fig.

To obtain the RUI, we excluded urban communes from 1992, as outlined in the administrative division published in the official bulletin of August 26, 1992, N4165, from the eVANUI-defined areas. This approach is particularly suitable for countries, where the transition zone between rural and urban areas is modestly developed, while the rural areas still lack certain basic infrastructure, particularly electricity.


$$eVANUI=NDVI*\left(1-HGNTL\right),$$ (5)

#### Built-up expansion and the impact on rural land

To assess the spatial change in the rural landscape, especially within the RUI due to urban sprawl, we used the LandTrendr spectral-temporal segmentation algorithm [65]. A variety of spectral indices as Tasseled Cap structures (TCB, TCG, TCA, TCW), the NDVI, and the Enhanced Vegetation Index (EVI), were tested to select the most sensitive one for detecting land conversion from vegetation to built-up in our study area. The LandTrendr algorithm is commonly used for detecting forest disturbances and recovery [66–68], and it has been increasingly adopted for various land cover change assessments, including changes in crop land [69], mining disturbance and restoration [70], and land conversion from vegetation to impervious surfaces [71].

This algorithm accurately identifies land disturbances based on spatial patterns of land-cover change magnitude (spectral difference), year of disturbance, and duration of changes [68, 72]. However, it is imperative to emphasize the significance of scrutinizing time intervals, especially in studies centered around urban regions undergoing rapid expansion, due to the potential occurrence of disturbances between the selected intervals [22].

For validation process, we identified 120 areas of disturbance using the LandTrendr algorithm, and cross-referenced the year of each disturbance with historical high spatial resolution Google Earth Pro images from the corresponding years. This comparison served to confirm the accuracy of our results. According to [66], this approach is well suited for the continuous monitoring of land disturbance using moderate-resolution satellite imagery.

## Results

### Dynamics of built-up

#### Built-up cover in the 70s

**Fig 4:**
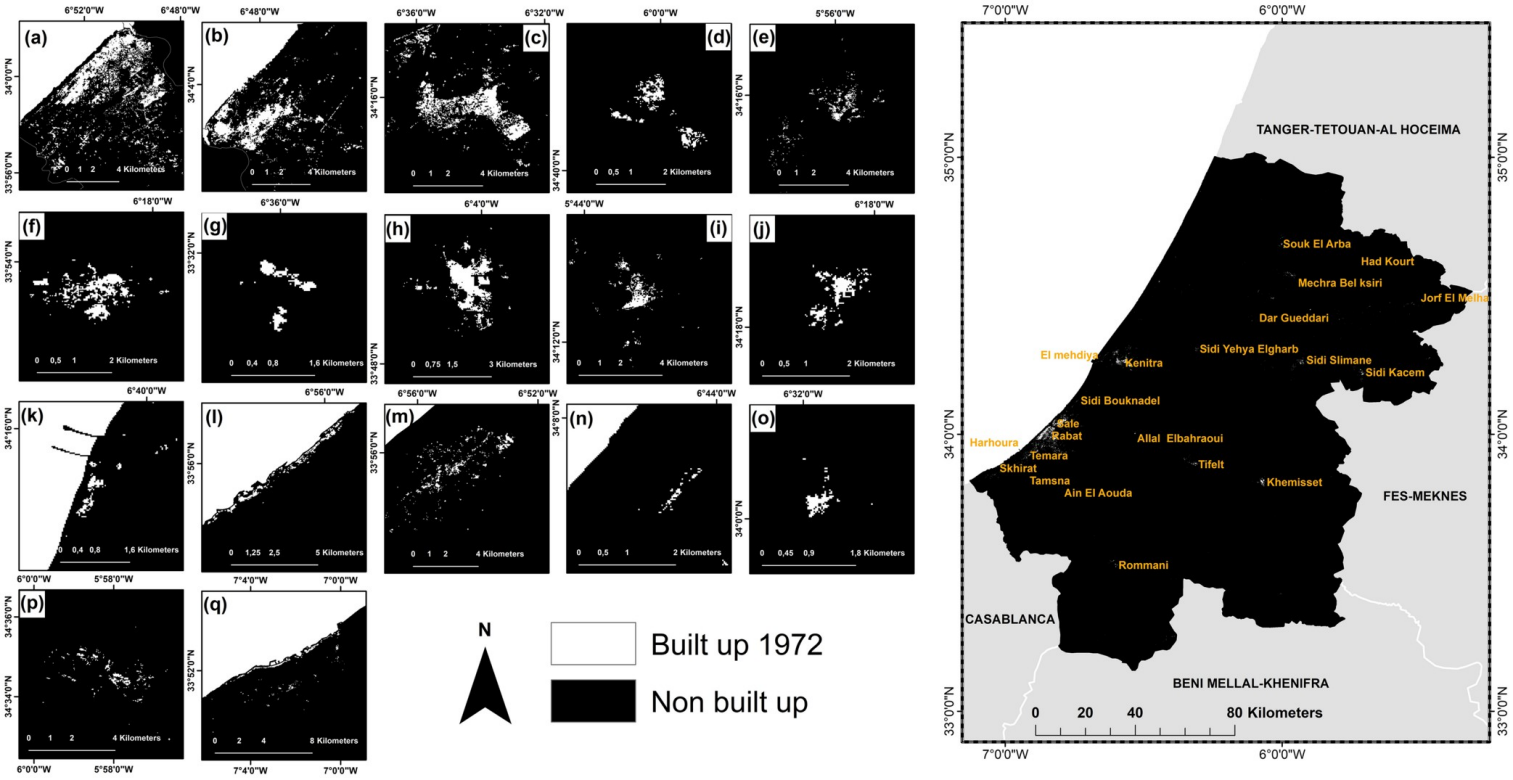
Random forest binary classification of the colorized Corona image for the RSK region, Morocco (1972). (a) Rabat, (b) Sale, (c) Kenitra, (d) Souk El Arba, (e) Sidi Slimane, (f) Tifelt, (g) Rommani, (h) Khemisset, (i) Sidi Kacem, (j) Sidi Yehya, (k) El Mehdiya, (l) Harhoura, (m) Temara, (n) Sidi Bouknadel, (o) Sidi Allal Elbahraoui, (p) Machra belksiri, (q) Skhirate. Map was created using ArcGIS (version 10.6) from Esri (http://www.arcgis.com).

In 1972, the built-up in the study area covered a surface of 63.4 km^2^ or about 0.4%, whereby 17 urban agglomerations of different sizes along with several small scattered clusters were classified; Rabat, Sale, Kenitra, Souk El Arba, Sidi Slimane, Tifelt, Roumani, Khemisset, Sidi Kacem, Sidi Yehya, El Mehdiya, Harhoura, Temara, Sidi Bouknadel, Sidi Allal Elbahraoui, Machra belksiri, and Skhirate (Fig 4).

The three largest cities Rabat, Sale, and Kenitra, occupied 21.9 km^2^, 11.3 km^2^, and 11.0 km^2^, respectively, medium and small agglomerations such as Khemissate, Tiflet, Sidi Yehya, Souk El Arba, Sidi Kacem, Temera, Sidi Slimane, Sidi Allal Elbahraoui, and Skhirate, had areas of less than 3.5 km^2^ each.

#### Built-up cover between 1985 and 2020

**Fig 5:**
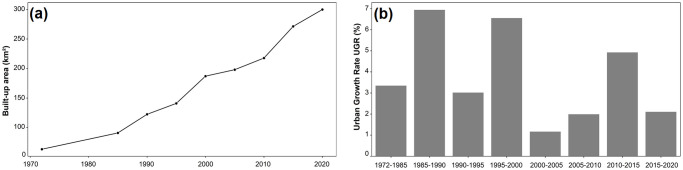
Evolution of the urban fabric in the RSK region, Morocco from 1972 to 2020. (a) Urban area (km^2^), (b) urban growth rate (UGR %). Map was created using ArcGIS (version 10.6) from Esri (http://www.arcgis.com).

During 1985–2020, the use of Landsat archives at 5-year intervals yielded eight binary images of the urban built-up fabric. Their validation images showed an OA ranging from 88–95%, Table 3. The random forest classifications, which met the evaluation criteria for land cover classifications, were more accurate than two well-known global open-access datasets: the Global Human Settlement Layer (GHSL) and the DLR World Settlement Footprint (WSF), S4 Fig. Our maps indicated a three-fold increase of the built-up fabric from 1985 to 2020 (Fig 5). This increase in the size of the agglomeration was accompanied by an increase in the number of agglomerations from 17 to 23.

**Table 3:** The overall accuracy of the binary RF classifications of the RSK region, Morocco from 1985 to 2020.

Satellite	RF classification results per year	OA (%)
**Landsat 5**	1985	88
	1990	93
	1995	93
	2000	95
	2005	94
	2010	92
**Landsat 8**	2015	90
	2020	95

The AUE ranged from 2.1 km^2^ (1972–1985), 6.3 (1985–1990), 3.7 (1990–1995), 9.2 (1995–2000), 2.2 (2000–2005), 3.9 (2005–2010), 10.7 (2010–2015) to 5.7 km^2^ (2015–2020).

**Fig 6:**
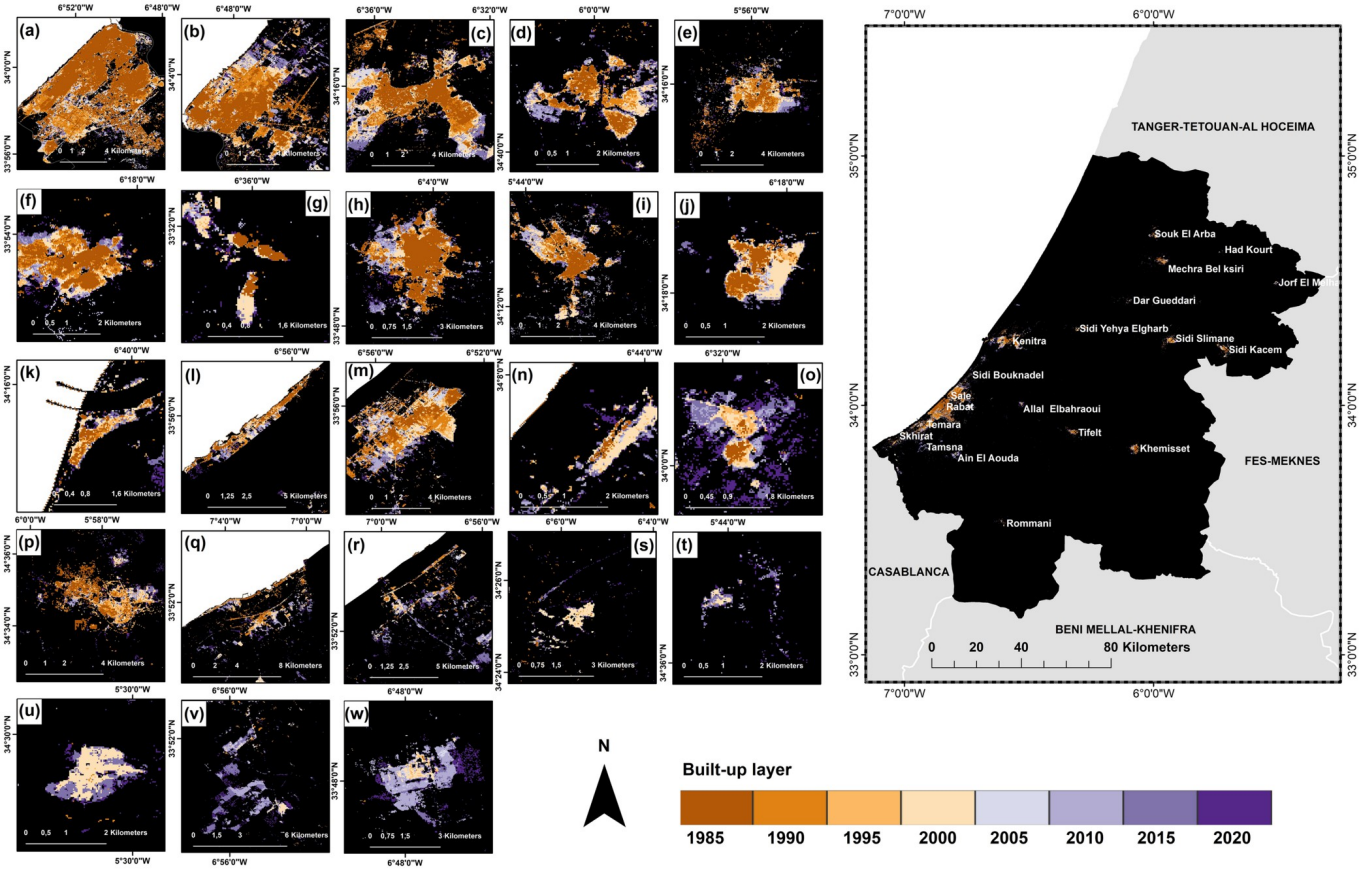
Spatial evolution of the built-up area per city, in the RSK region, Morocco from 1985 to 2020. (a) Rabat, (b) Sale, (c) Kenitra, (d) Souk El Arba, (e) Sidi Slimane, (f) Tifelt, (g) Rommani, (h) Khemisset, (i) Sidi Kacem, (j) Sidi Yehya, (k) El Mehdiya, (l) Harhoura, (m) Temara, (n) Sidi Bouknadel, (o) Sidi Allal Elbahraoui, (p) Machra belksiri, (q) Skhirate, (r) Ain Attik, (s) Had Kourt, (t) Dar Gueddari, (u) Jorf El Melha, (v) Tamsna, (w) Ain El Aouda. Map was created using ArcGIS (version 10.6) from Esri (http://www.arcgis.com).

**Fig 7:**
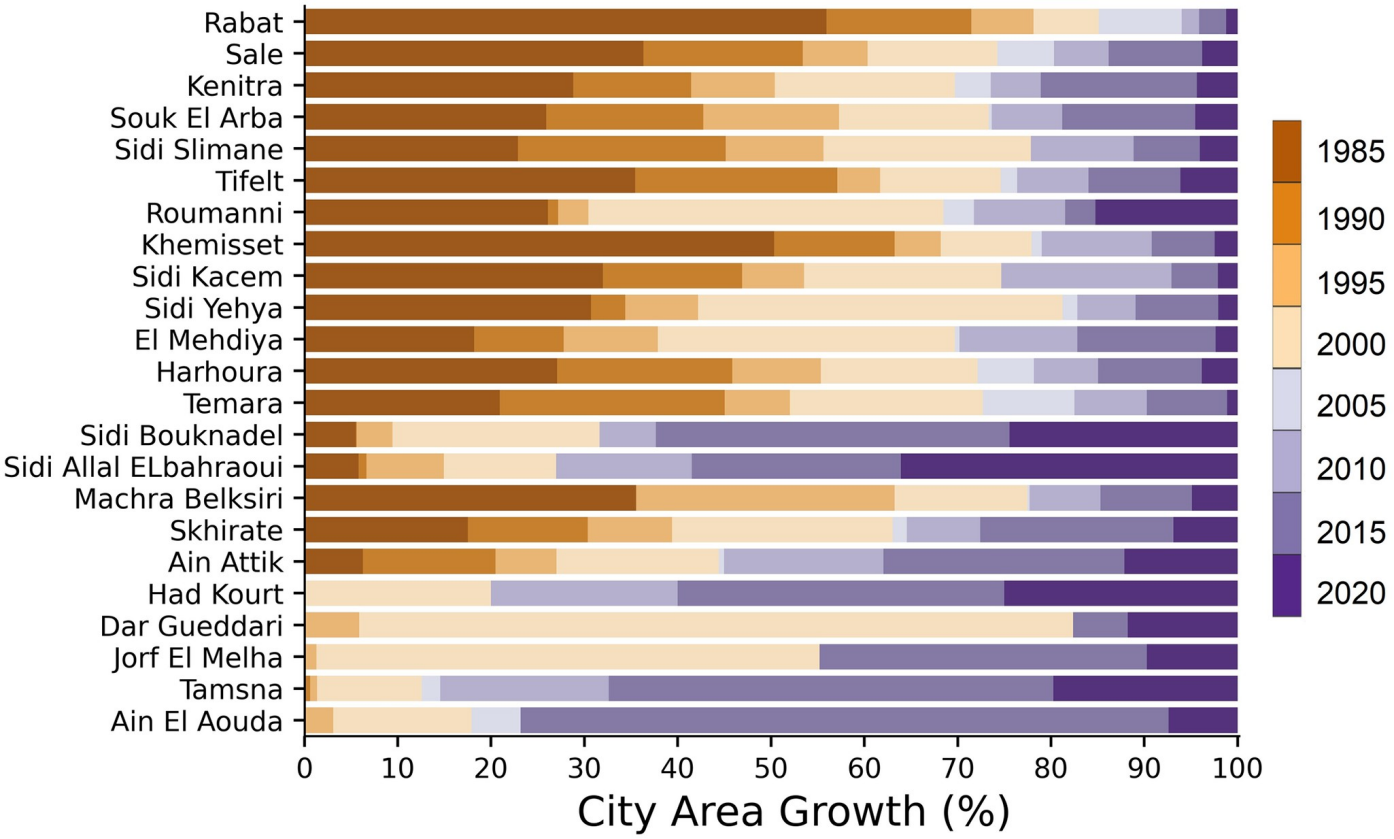
Built-up cover growth per city (%), in the RSK region, Morocco from 1972 to 2020.

**Fig 8:**
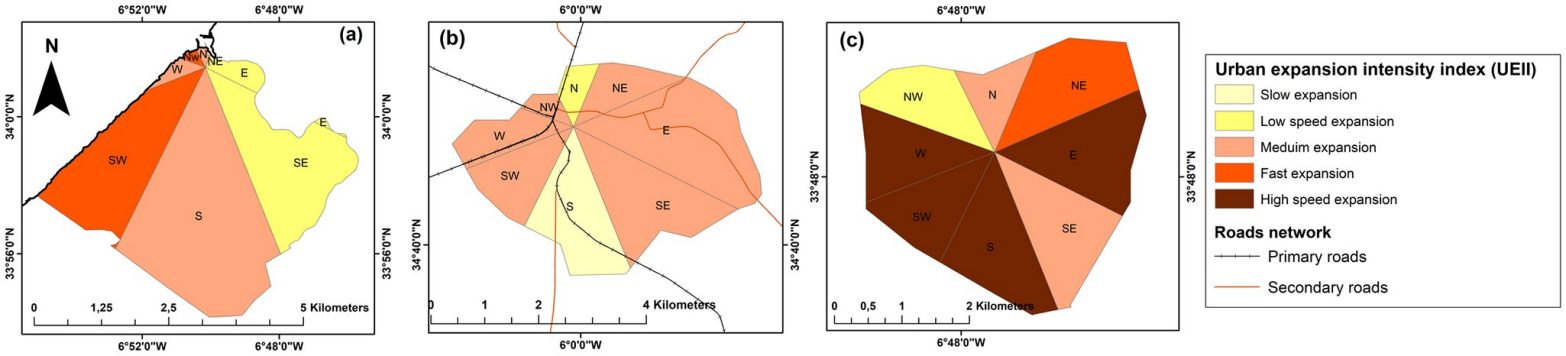
Urban expansion intensity in three different cities of the RSK region, Morocco from 1972 to 2020. (a) Rabat (metropolitan city), (b) Souk El Arba (medium city), and (c) Ain El Aouda (new city). Map was created using ArcGIS (version 10.6) from Esri (http://www.arcgis.com).

**Fig 9:**
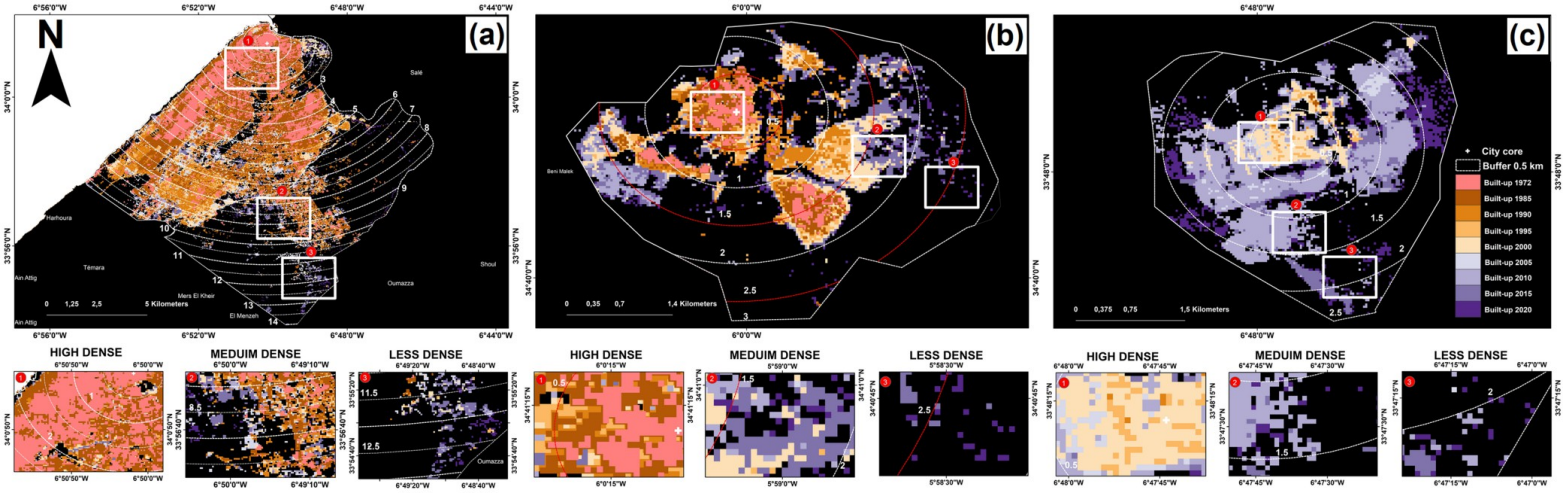
Urban growth from the city center to its boundary, at a 0.5 km interval buffer for three different cities in the RSK region, Morocco from 1972 to 2020. (a) Rabat, (b) Souk El Arba, and (c) Ain El Aouda. Map was created using ArcGIS (version 10.6) from Esri (http://www.arcgis.com).

Urban growth affected each city to varying degrees and levels, with a percentage of expansion fluctuating over time (Fig 6). The largest cities in the region, Rabat, Kenitra, and Sale, experienced the most significant changes to their urban fabric especially between 1985 and 1990 (Fig 7). These cities expanded outward in various directions and at varying rates, particularly toward the coastline, where the observed UEII was higher than in other directions. Small to medium-sized towns, such as Souk El Arbaa, developed along the main transport route based on the accessibility of the land. For new towns created around major cities to absorb demographic and urban pressure, such as for the city of Ain El Aouda, urban expansion was extremely rapid and multidirectional (Fig 8). Regardless of the urban growth pattern in the RSK cities, urban expansion density was always high in the city center, while it was less dense on the citys’ outskirts (Fig 9).

### Rural-urban interfaces

**Fig 10:**
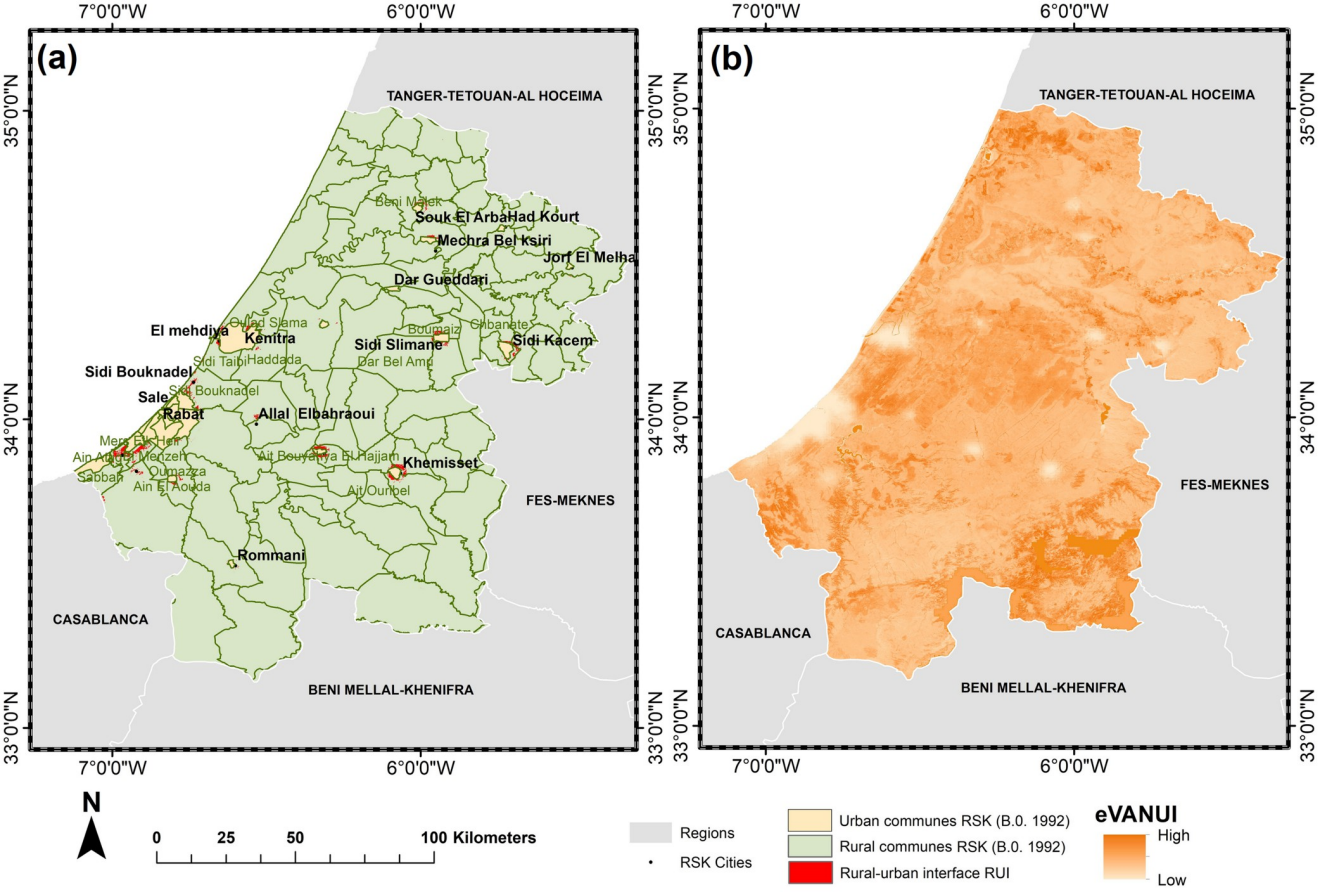
The limits of urban and rural interfaces in the RSK region, Morocco. Map was created using ArcGIS (version 10.6) from Esri (http://www.arcgis.com).

Most major towns in the RSK region have expanded into rural communities where the urban fabric meets rural space and producing mixed-identity transitory interfaces that are not fully urban yet no longer fully rural. Coupling the eVANUI with the 1992 administrative division allowed the mapping of eleven primary transition interfaces covering a total area of 113.6 km^2^ in the RSK region since 1992 (the year when nighttime products became available) (Fig 10).

In the case of Rabat, a small transitional RUI has emerged at the southern boundary towards the north of El Menzah, while the majority of the city’s urban fabric has spread horizontally towards Temara, forming an urban corridor connecting the two towns and subsequently extending away from Temara’s urban boundary towards the former rural commune of Ain El Aouda. Similarly, a second urban growth area has formed around Skhirate, expanding southward to the rural commune of Sebbah and southeast to the former rural commune of Ain Attik.

Salé’s urban fabric has also extended since 1995 to incorporate the rural commune of Sidi Bouknadel on the city’s northeastern outskirts, establishing a new RUI. For Kenitra, urban development began in 1995 beyond its borders, forming an RUI pattern beyond the city’s boundaries to the southeast and southwest, towards Oulad Salama, Haddada, and Sidi Taibi rural communes.

In addition, Tifelt’s urban fabric has gradually evolved in the southeast section of Ait Bou Yahya Elhajjama since 2010, creating a new RUI outside the city. Khemisset’s urban expansion resulted in the development of an RUI in the south, towards the rural commune of Ait Ouribel. Sidi Slimane’s urban fabric expanded to the southwest and north directions, forming a transition zone in the surrounding rural communes (Boumezz, Dar El Amri).

Similarly, Sidi Kacem grew beyond its urban borders in 1995 into Chbanat, forming a transitional fringe between urban and rural territory. The urban fabric of Souk El Arba has also expanded east and west towards the rural commune of Benni Malek, since 2000, forming an RUI in both directions. Recently, the reclassified cities of Ain El Aouda and Sidi Allal Elbahraoui have also formed a RUI pattern towards the rural communes of Oumazza and Ait Malek respectively.

#### Urban growth patterns and spatial changes on rural lands within the rural-urban interface

**Fig 11:**
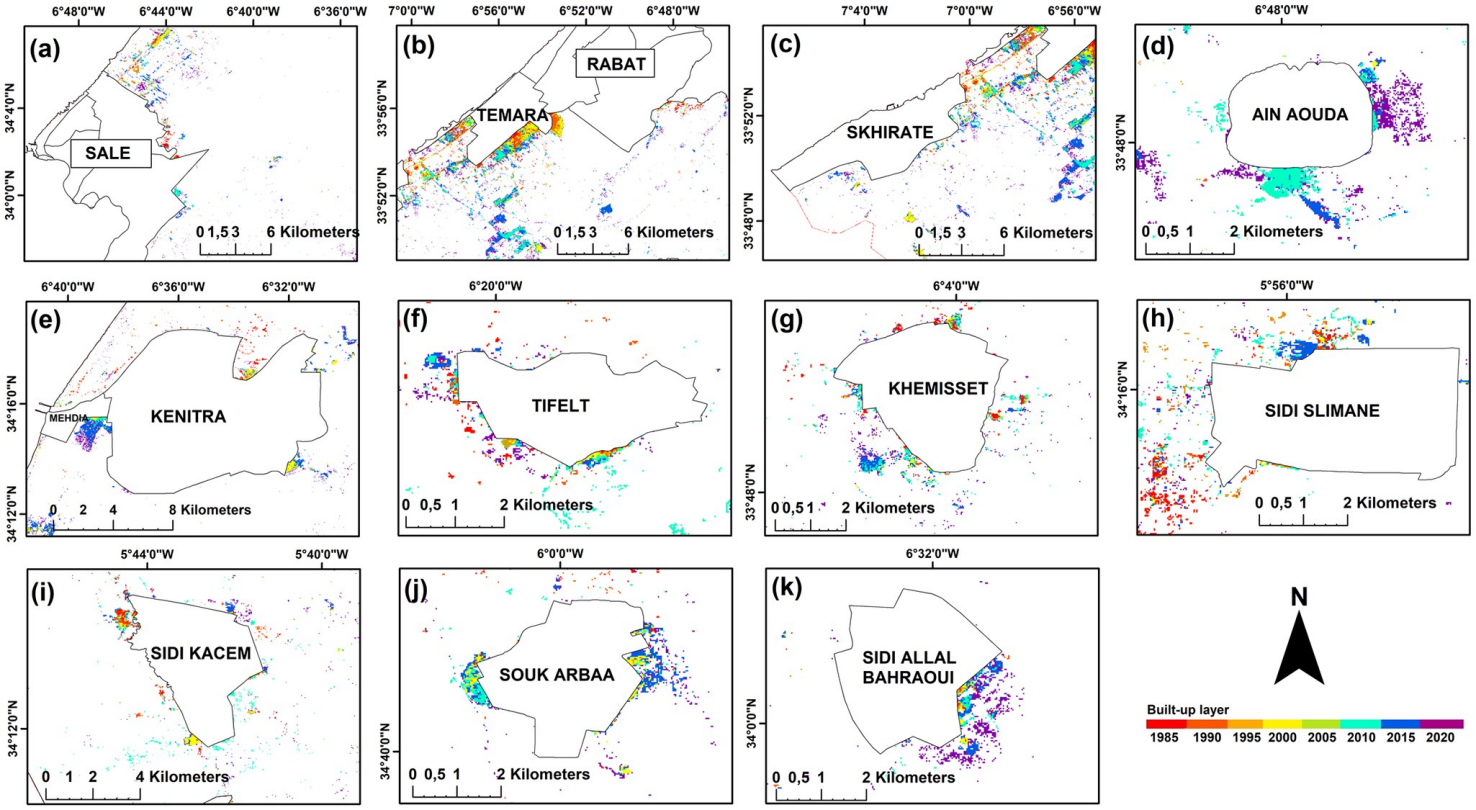
The development of the urban fabric inside the rural-urban interface from 1992 to 2020 in the RSK region, Morocco. (a) Sale, (b) Rabat, (c) skhirate, (d) Ain El Aouda, (e) Kenitra, (f) Tifelt, (g) Khemisset, (h) Sidi Slimane, (i) Sidi Kacem, (j) Souk El Arba, (k) Sidi Allal Elbahraoui. Map was created using ArcGIS (version 10.6) from Esri (http://www.arcgis.com).

**Fig 12:**
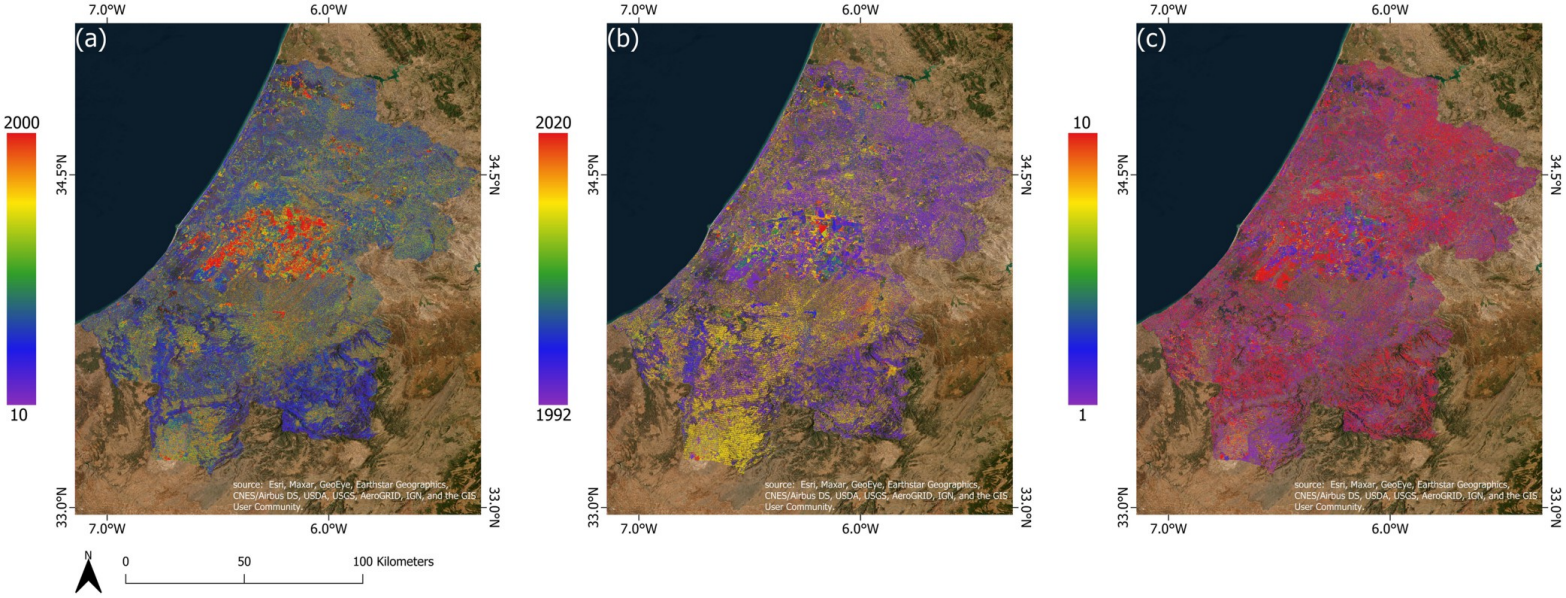
The LandTrendr results using the Tasseled Cap Brightness (TCB) in the RSK region, Morocco. (a) The magnitude of the change, (b) the year of the change, (c) the duration of the change. Map was created using ArcGIS (version 10.6) from Esri (http://www.arcgis.com). Basemap satellite images accessed from World Imagery Esri Tile Layer. Credits: Esri, Maxar, GeoEye, Earthstar Geographics, CNES/Airbus DS, USDA, USGS, AeroGRID, IGN, and the GIS User Community.

**Fig 13:**
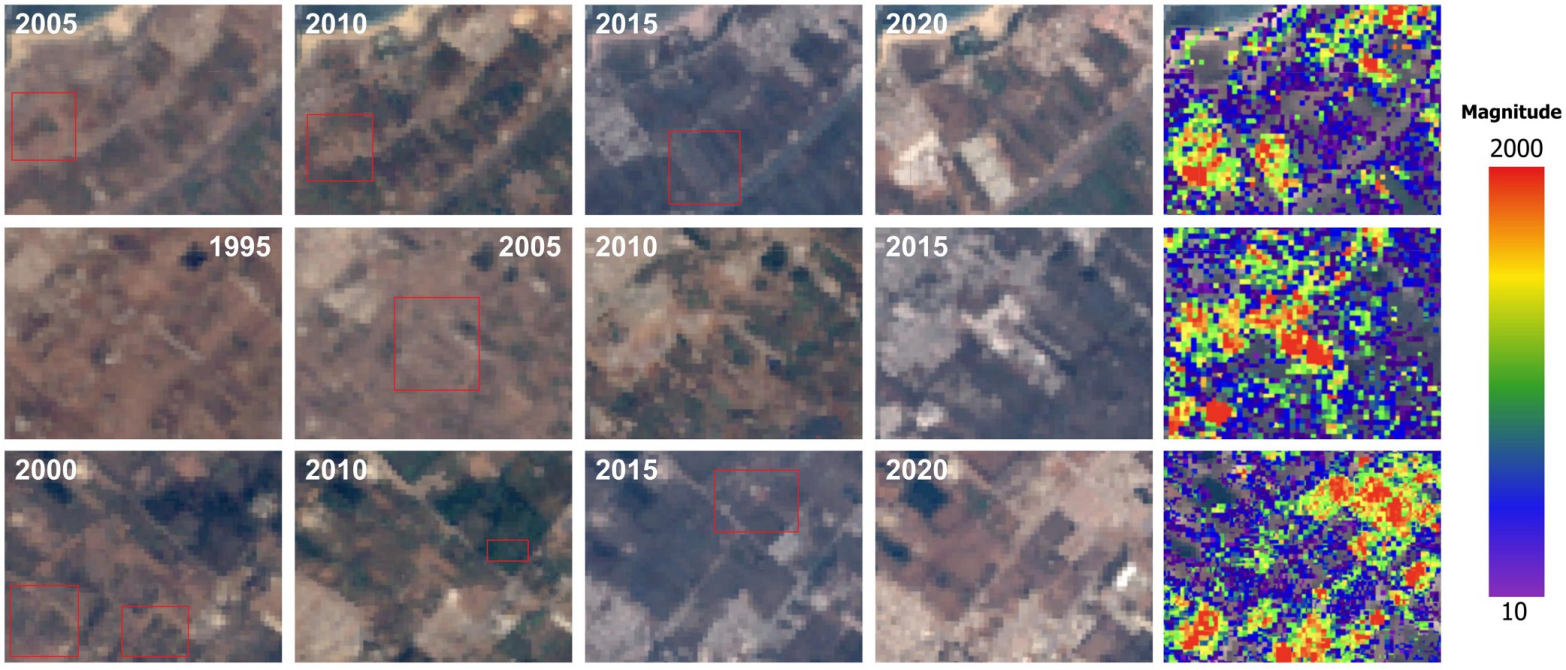
Example of some rural lands, impacted by the urban expansion inside the rural-urban interfaces of Rabat and Sale, Morocco. Landsat images were obtained from USGS Earth Explorer (https://earthexplorer.usgs.gov).

The built-up area that formed inside these interfaces was 3.4 km^2^ in 1992 and expanded to 22.5 km^2^ in 2020. The nature of these built-up areas in the rural-urban interface differs from rural built-up areas because they are linked and relatively close to one another, and they are located on the edge of urban communes, in contrast to the rural settlements which were always very scattered (Fig 11). The temporal segmentation LandTrendr algorithm, utilizing the overall brightness of the land surface as a crucial indicator of change, revealed that disturbances in the region began in the early years of the prospection and continued with varying intensities from area to area until 2020 (Fig 12). Most of the changes were of low magnitude, except for the RUIs around major cities and in the region’s center, where the magnitude was significantly higher. The coincidence of a strong disturbance (high change magnitude) with an abrupt change in duration in the RUIs of coastal and central cities indicated the emergence of newly converted areas. The validation of LandTrendr results using the TCB index in various rural-urban areas confirmed that in many high-magnitude/low duration areas within the RUIs, the urban fabric process had been expanded on many rural lands including vegetation lands (Fig 13).

## Discussion

The results of analyzing high-resolution historical grayscale satellite images and Landsat series of moderate spatial resolution showed that the RSK region experienced rapid urban expansion that changed the structure and shape of all the major cities in the region. The built-up surface expanded horizontally, with an annual UGR of 7.8%. This rate was lower compared to the majority of African and Asian countries, such as Kenya and (29.3%) and Pakistan (29.0%), but higher than that observed in the United States and Japan, where UGRs ranged from 2 to 4% [73].

The evolution of built-up areas in most RSK cities was highly correlated with the coastline and road network. Cities along the Kenitra-Temara axis developed considerably faster than other cities in the region. The increasing coastal urbanization in the region was mainly due to the numerous advantages and opportunities for resource-based economic activity that the coastline offers [74]. The intensity of growth in the central cities varied depending on their proximity to road infrastructure, which offers economic vitality and greater accessibility. Many studies have confirmed the strong effect of the road networks on urban growth patterns [75]. The spatial transformations occurred in several phases and were primarily influenced by environmental, social, political, and economic forces.

### Driving forces

During Morocco’s colonial period (1912–1956), a profound transformation of the social and urban structures occurred as attention shifted from Fez, the former capital, to Rabat, the new administrative capital and to the coastal regions due to their high economic importance [76, 77]. Consequently, rapid urbanization took place within these key hubs, driven by the concentration of political, economic, and administrative activities and the interplay between natural population growth and rural migration seeking economic opportunities and improved living conditions in these centers. The expanding population in urban areas, created a surge in demand for housing, infrastructure, and services, fundamentally altering the dynamics of the capital region, fueling the urban expansion and gradually shaping the urban landscape.

The 1970s marked the beginning of political and social stability following the transition from colonial rule to independence. The population began to rapidly grow as documented by the United Nations Population Division. The overall number of urban agglomerations in RSK increased to 17, including both large cities and small agricultural-based settlements such as Khemisset and Tifelt.

In the mid-1980s, Morocco suffered from four consecutive years of drought, which significantly set back the country’s development [78], particularly in the RSK region, which is one of the primary rainfed agricultural regions in the country [40]. Given that Morocco’s economy heavily relies on agriculture [79], the stagnation in this sector due to drought had ripple effects on all other economic activities. Consequently, the pace of urban expansion considerably slowed down during this period. The UE between 1972 and 1985 was at most 2.1 km^2^ per year, growing from 63.3 km^2^ in 1972 to 90.9 km^2^ in 1985.

Between 1985 and 1995, Morocco experienced new socio-spatial dynamics and economic recovery. The country began to see the benefits of reforms and development projects including the structural adjustment programs (SAPs) [80], and the initiation of privatization [81]. These reforms actively promoted private sector participation in urban development across Morocco, including the RSK region, given its significance. Consequently, this period marked the beginning of the diffusion phase of the built-up fabric in the region with an annual urban growth rate of 5.5%. The privatization was reported in various countries as having significant effects on the demographic profile of cities and the emergence of urban migration patterns [82].

From 1995 to 2005, urbanized surfaces evolved in the region’s largest and medium-sized cities. This particular period witnessed a significant transformation in reconstruction policies, marked by a focus on promoting investments in the construction sector and the introduction of a program known as "Cities without Slums", which aimed to eliminate informal settlements through the establishment of affordable economic housing primarily particularly on the outskirts of urban centers [83]. Consequently, a process of peri-urbanization began to increase in the outskirts of the largest and medium cities at the expense of surrounding rural communes. The annual UGR within the RUIs during this period, reached 14.4%.

Between 2005 and 2010, the country adopted a stringent regulatory strategy to control the expansion of large urban centers, alleviate existing dysfunctions, and address urban development challenges. This approach involved the establishment of new cities to curb the chaotic expansion of the suburbs [84], and the reclassification of some rural communes as urban such ad Ain Attik (2009), and Sidi Allal Elbahraoui (2009). The government allocated a substantial budget to these newly established urban centers, to develop the necessary infrastructure. As a result, there was a notable impact on territorial development, leading to a decrease in the annual UGR within the RUI to 8% during this period. Conversely, the built-up coverage within the newly formed urban centers experienced an increase (Fig 7).

From 2010 to 2020, the overall scenario exhibited minimal changes, with the UGR increase, especially within the new urban centers, as a result of the implementation of road infrastructure development programs [42] and a decrease in interest rates on housing loans. However, the annual UGR within the RUI remained stable at 8%, similar to the previous period. According to the national reports, the reclassified cities have partially alleviated the pressure on the suburbs of major cities within a period before developing also an interface outside their administrative boundaries (Fig 11). Nonetheless, the satellite cities have encountered significant challenges due to deficiencies in essential infrastructure since their inception [85].

### Effects on rural hinterlands

The continuous horizontal urban growth resulted in the alteration of the rural landscape in the outskirts of metropolitan centers. Our study revealed that the extensive and haphazard urban sprawl came mainly at the expense of rural vegetation and some open lands in the RUI. This pattern of change is similar to that seen in many other countries of the Global South that have experienced a significant loss of rural vegetation due to urban sprawl [86–88]. This is often due to a scarcity of strategies to control unstructured urban sprawl and a lack of programs focused on promoting sustainable development in both the urban core and rural areas.

## Conclusions

Our study approach using grayscale and Landsat satellite archives diverged from traditional techniques in urban land use change studies by taking into account not only the spatio-temporal evolution of the built-up area within urban boundaries, but also the alterations in the rural landscape at the rural-urban fringe of major cities. The results showed that the expansion of urban development in RSK has been rapid and unbalanced. This resulted in a significant impact on the rural landscapes, including vegetation lands in the periphery of major coastal and central cities. Despite efforts to regulate urban growth, mitigate its effects, promote rural development, and conserve natural resources, urban planning in Morocco still requires greater emphasis on the environmental dimension of rural-urban transformation and subsequent urban sprawl.

## Supporting information

S1 Fig Learning-based colorization of grayscale Corona satellite image. (a) The grayscale Corona image, CORONA Satellite image courtesy of the U.S. Geological. Survey (DS1117-1011DF072—DS1117-1011DF086, 26.05.1972) (https://earthexplorer.usgs.gov). (b) The colorized image generated by the GAN model. Map was created using ArcGIS (version 10.6) from Esri (http://www.arcgis.com).
(TIF)

S2 Fig Datasets used to calculate the eVANUI. (a) The mean of the NDVI image collection from 1992 to 2020. (b) The median of Harmonized Global Night Time Lights (HGNTL) collection from 1992 to 2020. HGNTL reprinted from Xuecao Li et al [43] under a CC BY 4.0 International license (https://gee-community-catalog.org/projects/hntl/). Map was created using ArcGIS (version 10.6) from Esri (http://www.arcgis.com).
(TIF)

S3 Fig Delineation of rural urban interface. (a) The limits of functional and nonfunctional areas. (b) The extracted rural urban interface. Map was created using ArcGIS (version 10.6) from Esri (http://www.arcgis.com).
(JPG)

S4 Fig Comparison of the random forest classification of the built-up with two open access products GHSL2000 and WSF2015. Landsat images were obtained from the USGS Earth Explorer (https://earthexplorer.usgs.gov). GHSL data was provided by the European Commission, Joint Research Centre (JRC) (http://data.europa.eu/89h/jrc-ghsl-ghs_built_ldsmt_globe_r2015b), under a CC BY 4.0 International license (http://creativecommons.org/licenses/by/4.0/). WSF data was provided by the Earth Observation Center (EOC) at the German Aerospace Center (DLR) (https://www.un-spider.org/links-and-resources/data-sources/world-settlement-footprint-2015-wsf-dlr-eoc), under a CC BY 4.0 International license (http://creativecommons.org/licenses/by/4.0/). Map was created using ArcGIS (version 10.6) from Esri (http://www.arcgis.com).
(JPG)
