# Supplementary figures and images for: Monitoring rural-urban transformation in the coastal region of Rabat-Sale-Kenitra, Morocco
Source: PLoS One. 2023 Aug 31;18(8):e0290829. doi: 10.1371/journal.pone.0290829 (PMC10470892; doi:10.1371/journal.pone.0290829)

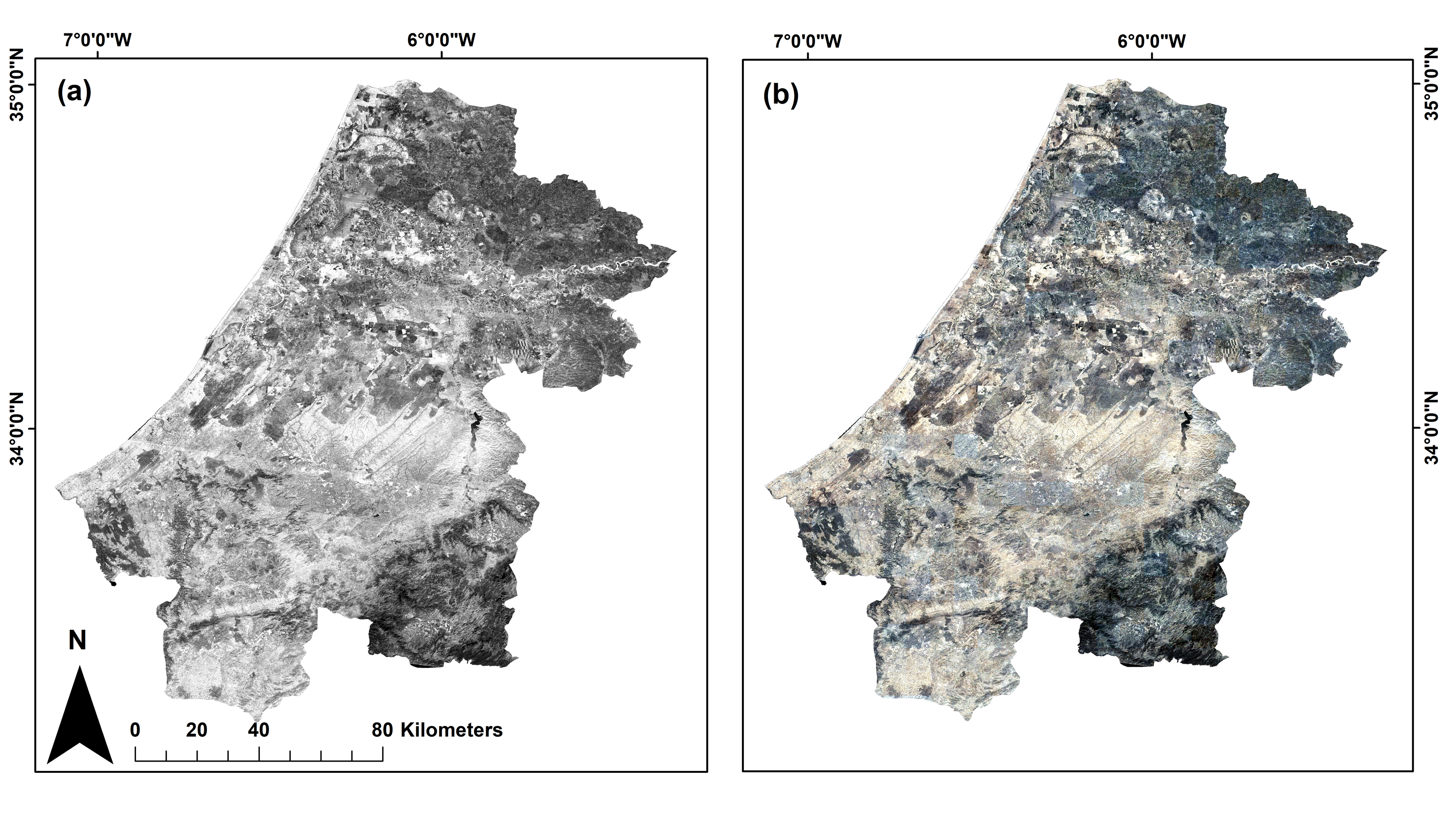

Supplement: S1 Fig — (a) The grayscale Corona image, CORONA Satellite image courtesy of the U.S. Geological. Survey (DS1117-1011DF072—DS1117-1011DF086, 26.05.1972) (https://earthexplorer.usgs.gov). (b) The colorized image generated by the GAN model. Map was created using ArcGIS (version 10.6) from Esri (http://www.arcgis.com). (TIF) [file pone.0290829.s001.tif]

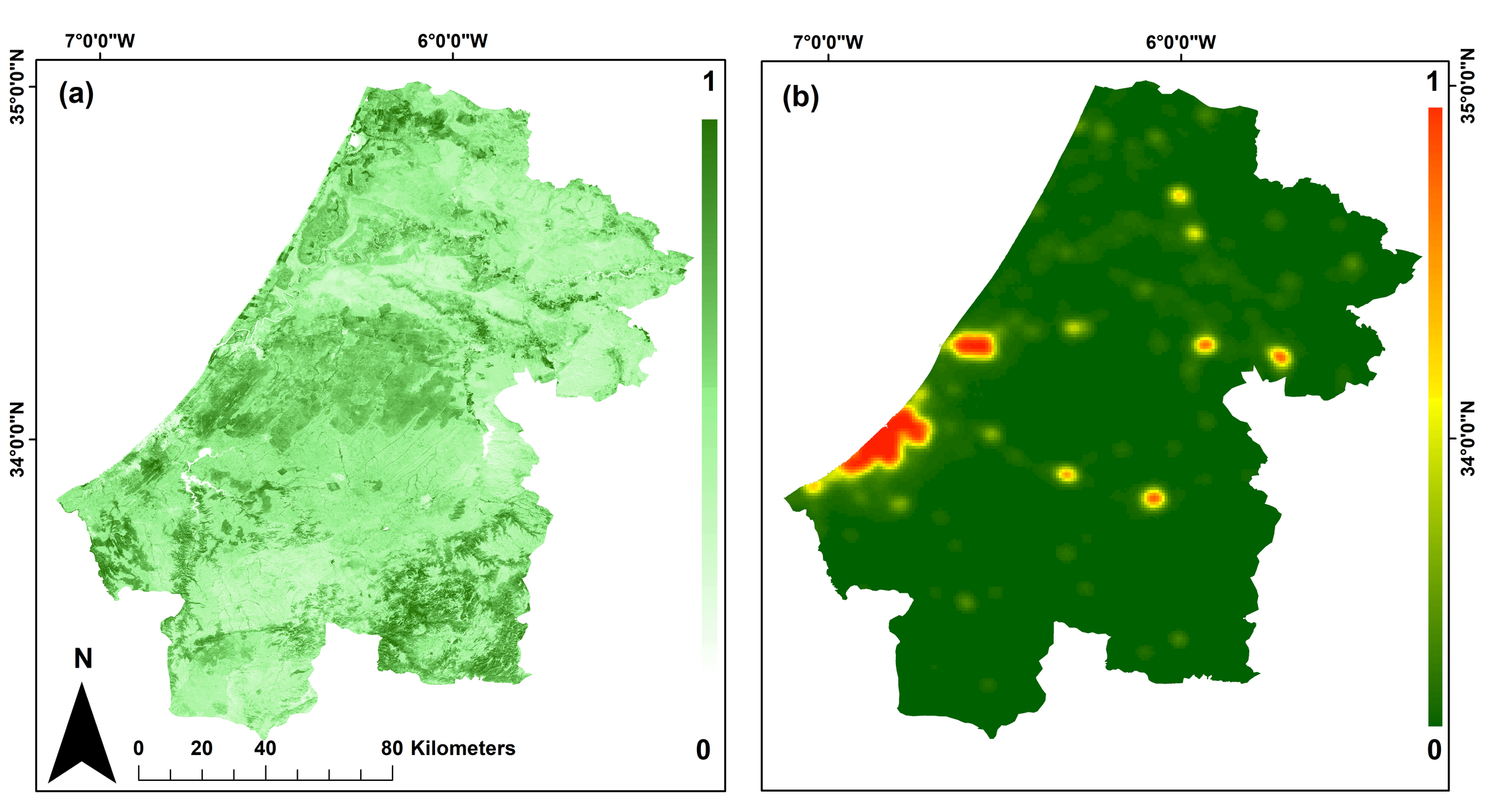

Supplement: S2 Fig — (a) The mean of the NDVI image collection from 1992 to 2020. (b) The median of Harmonized Global Night Time Lights (HGNTL) collection from 1992 to 2020. HGNTL reprinted from Xuecao Li et al [43] under a CC BY 4.0 International license (https://gee-community-catalog.org/projects/hntl/). Map was created using ArcGIS (version 10.6) from Esri (http://www.arcgis.com). (TIF) [file pone.0290829.s002.tif]

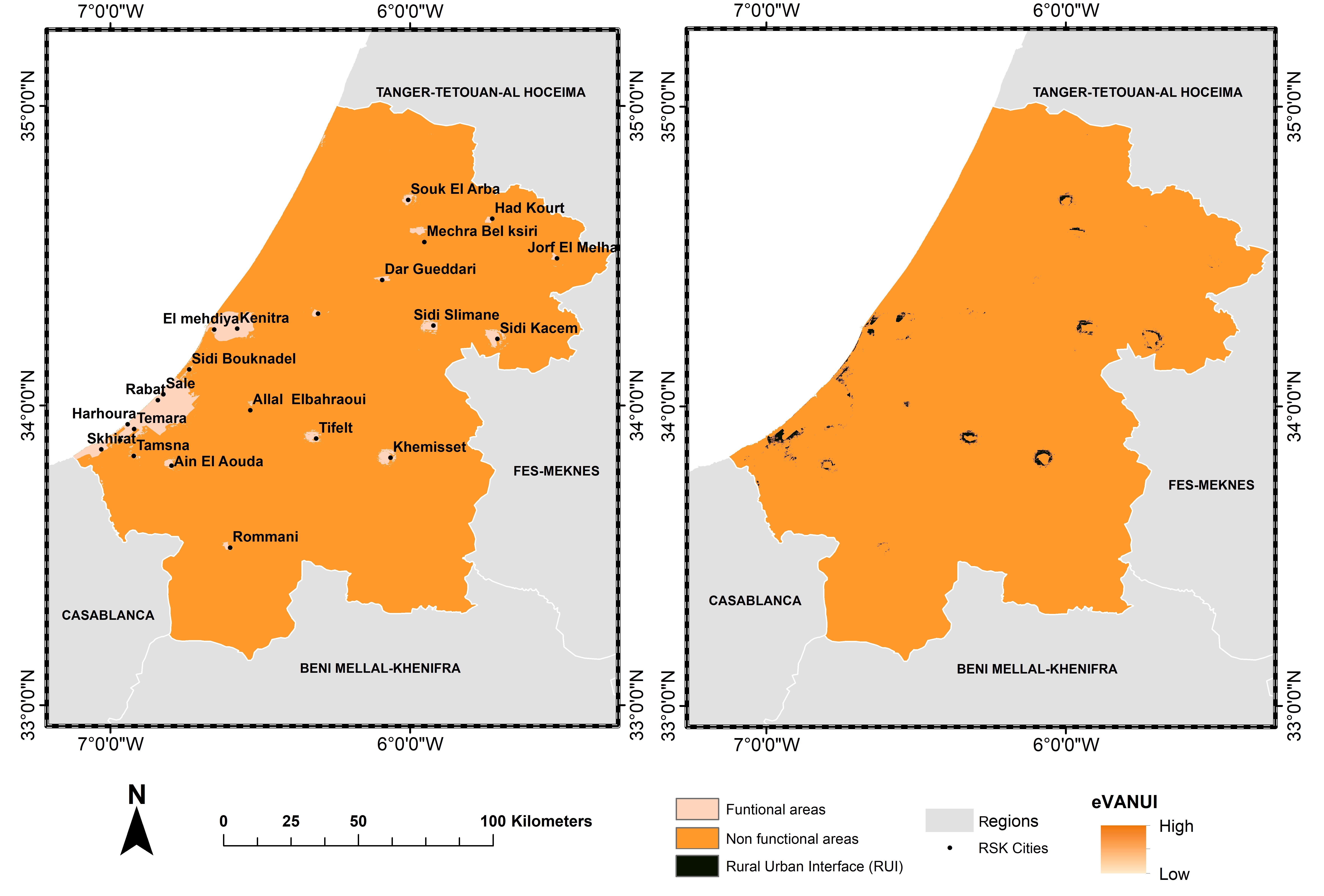

Supplement: S3 Fig — (a) The limits of functional and nonfunctional areas. (b) The extracted rural urban interface. Map was created using ArcGIS (version 10.6) from Esri (http://www.arcgis.com). (JPG) [file pone.0290829.s003.jpg]

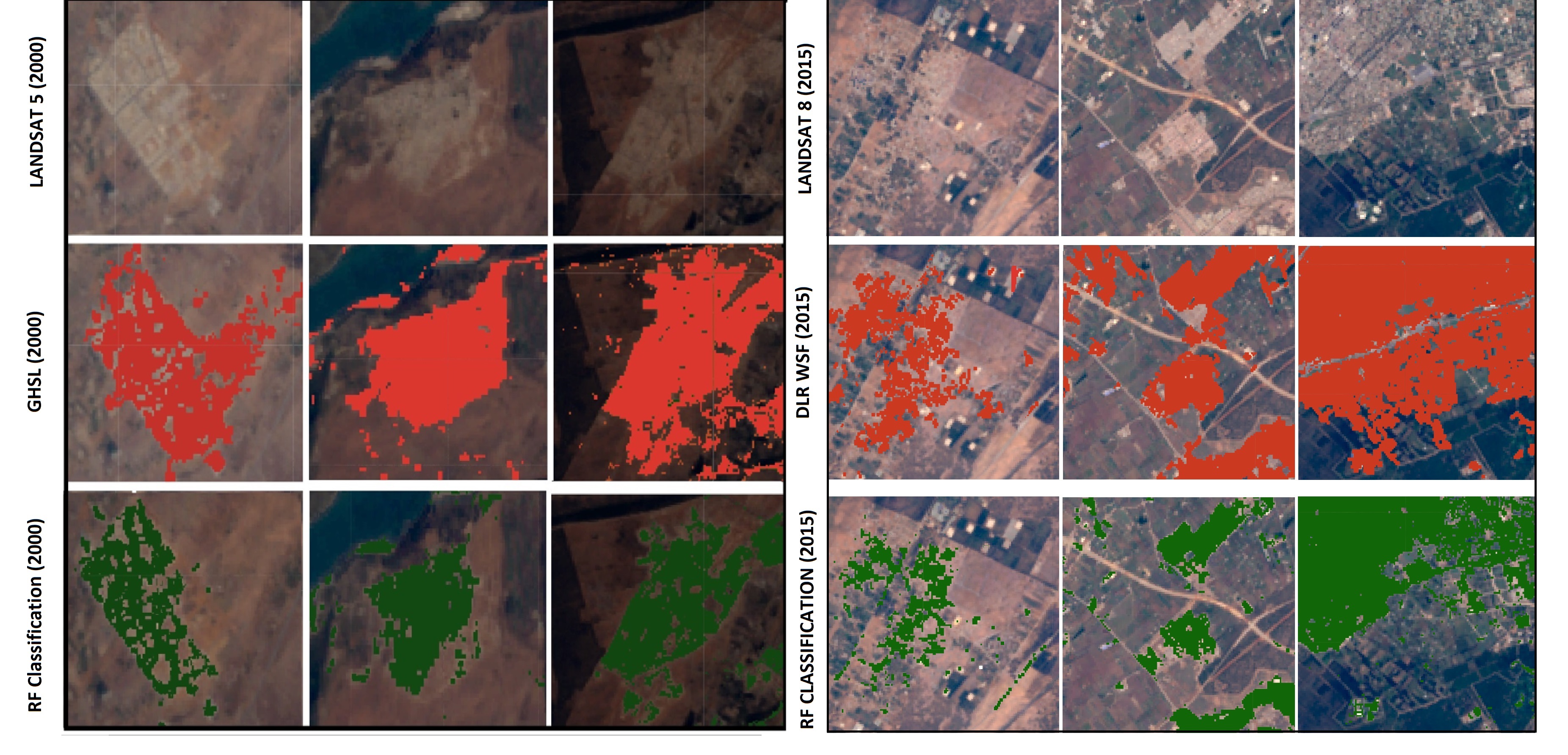

Supplement: S4 Fig — Landsat images were obtained from the USGS Earth Explorer (https://earthexplorer.usgs.gov). GHSL data was provided by the European Commission, Joint Research Centre (JRC) (http://data.europa.eu/89h/jrc-ghsl-ghs_built_ldsmt_globe_r2015b), under a CC BY 4.0 International license (http://creativecommons.org/licenses/by/4.0/). WSF data was provided by the Earth Observation Center (EOC) at the German Aerospace Center (DLR) (https://www.un-spider.org/links-and-resources/data-sources/world-settlement-footprint-2015-wsf-dlr-eoc), under a CC BY 4.0 International license (http://creativecommons.org/licenses/by/4.0/). Map was created using ArcGIS (version 10.6) from Esri (http://www.arcgis.com). (JPG) [file pone.0290829.s004.jpg]
